# Modeling the Tertiary Structure of the Rift Valley Fever Virus L Protein

**DOI:** 10.3390/molecules24091768

**Published:** 2019-05-07

**Authors:** Gideon K. Gogovi, Fahad Almsned, Nicole Bracci, Kylene Kehn-Hall, Amarda Shehu, Estela Blaisten-Barojas

**Affiliations:** 1 Center for Simulation and Modeling, George Mason University, 4400 University Drive, MSN 6A12, Fairfax, VA 22030, USA; ggogovi@masonlive.gmu.edu; 2 Department of Computational and Data Sciences, George Mason University, 4400 University Drive, MSN 6A12, Fairfax, VA 22030, USA; 3 School of Systems Biology, George Mason University, 10900 University Blvd., MSN 5B3, Manassas, VA 20110, USA; falmsned@masonlive.gmu.edu (F.A.); nbracci@masonlive.gmu.edu (N.B.); 4 National Center for Biodefense and Infectious Diseases, George Mason University, 10650 Pyramid Place, MS 1J5, Manassas, VA 20110, USA; 5 Department of Computer Science, George Mason University, 4400 University Drive, MSN 4A5, Fairfax, VA 22030, USA; 6 Department of Bioengineering, George Mason University, 4400 University Drive, MSN 1J7, Fairfax, VA 22030, USA

**Keywords:** Rift Valley fever virus, multidomain protein, tertiary structure, computational structure determination

## Abstract

A tertiary structure governs, to a great extent, the biological activity of a protein in the living cell and is consequently a central focus of numerous studies aiming to shed light on cellular processes central to human health. Here, we aim to elucidate the structure of the Rift Valley fever virus (RVFV) L protein using a combination of in silico techniques. Due to its large size and multiple domains, elucidation of the tertiary structure of the L protein has so far challenged both dry and wet laboratories. In this work, we leverage complementary perspectives and tools from the computational-molecular-biology and bioinformatics domains for constructing, refining, and evaluating several atomistic structural models of the L protein that are physically realistic. All computed models have very flexible termini of about 200 amino acids each, and a high proportion of helical regions. Properties such as potential energy, radius of gyration, hydrodynamics radius, flexibility coefficient, and solvent-accessible surface are reported. Structural characterization of the L protein enables our laboratories to better understand viral replication and transcription via further studies of L protein-mediated protein–protein interactions. While results presented a focus on the RVFV L protein, the following workflow is a more general modeling protocol for discovering the tertiary structure of multidomain proteins consisting of thousands of amino acids.

## 1. Introduction

The three-dimensional (tertiary) structure of a protein governs to a great extent its biological activity, as molecules employ their tertiary structures to complement and bind each other [1]. Due to the premise that a tertiary structure is key to obtaining a molecular-level understanding of cellular processes central to human health, resolving tertiary protein structures is a compelling research thrust in both dry and wet laboratories [2].

A collaborative project between our laboratories aims to better understand the replication of the Rift Valley fever virus (RVFV) and, in particular, the key role of the protein encoded by the L segment of the RVFV RNA. RVFV is an arbovirus in the Bunyavirales order, Phenuiviridae family, and Phlebovirus genus. RVFV was discovered in the Great Rift Valley of Kenya in 1931 [3]. Since that time, is has caused periodic outbreaks in human and livestock populations throughout Africa, and has even spread into the Arabian Peninsula. The virus is vectored by mosquitoes and, as such, outbreaks tend to follow periods of heavy rainfall that increase significantly mosquito populations [3].

RVFV causes Rift Valley fever (RVF), which has a wide range of clinical symptoms. A mild febrile illness is typically observed in humans. In some cases, this can progress to more severe illnesses, including liver disease, encephalitis, and hemorrhagic fever. Neurological issues are also observed, including paralysis, dizziness, headaches, delirium, vertigo, and hallucinations [4]. Ophthalmologic complications are observed in up to 10% of RVFV-infected humans, including photophobia and retinal hemorrhaging. Mortality is observed in 1–2% of people with severe disease [4]. RVFV causes more severe disease in sheep, cattle, and goats, with a mortality rate of 70% in young animals and 20–30% in adult ruminants. In addition, infected pregnant animals undergo spontaneous abortion at an alarmingly high rate, in the range of 40–100% [5,6]. Based on its ability to cause morbidity and mortality, and its potential use as an agent of bioterrorism, RVFV is classified as a select agent by both the Center for Disease Control and Prevention (CDC) and U.S. Department of Agriculture (USDA). Unfortunately, there are no Food and Drug Administration (FDA) approved vaccines for human use, although some do exist for veterinary use [6]. Likewise, there are no therapeutics available to treat RVFV, making this an area in need of additional research.

RVFV is a negative-sense RNA virus that contains three segments of viral RNA, the S, M, and L segments [7]. Most pertinent to our work is the L segment, which encodes only one protein, the RNA-dependent RNA polymerase (RdRp), referred to simply as the L protein. The L protein is responsible for viral mRNA transcription and for viral genomic replication, and it contains an endonuclease domain within its N-terminus region [8] that is critical for cap-snatching, where it cleaves 5${}^{\prime }$ m7G caps from host mRNAs. These host mRNAs are subsequently used as primers for viral transcription and to prevent viral RNA from triggering the innate immune response [7,9]. The L protein also contains an RdRp domain within its middle region, which is a domain found in all RNA viral polymerases to enable the production of viral RNA. The RVFV N protein (NP) coats the viral RNA to form the nucleocapsid, and this structure is needed for efficient viral replication [10]. RdRp domains have several conserved regions, one of which, termed motif C, contains SDD amino acids [11,12]. The mutation of these residues to GNN results in the loss of RVFV L protein transcriptional/replication activity [13], thus highlighting the significance of this region. Beyond its interaction with the RVFV NP, the only other protein–protein interaction identified to date for the L protein is the ability of forming L protein–L protein dimers. The interactions sites for L–L oligomers were narrowed down to the N-teminus (aa 1-222) and C-terminus (1219-2092) regions of the protein [13].

Currently, there is no structural information available for the RVFV L protein, either from transmission electron cryomicroscopy (Cryo TEM) or nuclear magnetic resonance (NMR), largely due to its size posing challenges to wet-laboratory efforts. Having a structural model of the RVFV L protein, however, is critical, as it would enable greater molecular knowledge about viral propagation, including the analysis of protein–protein and protein–RNA interactions that mediate viral replication and transcription. In addition, as antivirals are classically developed to target viral enzymes including polymerases [14], a structural model would facilitate drug-development efforts. For these reasons, this paper focuses on elucidating physically realistic tertiary structures comprising the native state of the L protein. The computational investigation challenge resides in the RVFV L protein’s large size and multiple-domain construct. To date, there are no modeling methods or protocols for holistically determining tertiary structures of multidomain proteins with long sequences of amino acids. Existing efforts are limited to assuming that the structure of the composing domains is known, which is not the case for the RVFV L protein. Indeed, some of the most interesting cases in viral biology involve multidomain proteins with little or no structural information at the domain level [15]. To make matters worse, the delineation of domain boundaries may also be unknown or uncertain.

In this work, we leverage various complementary computational approaches from the molecular-biology and bioinformatics domains to reconstruct, refine, and evaluate several physically realistic atomistic structural models of the L protein. Via a molecular-dynamics simulation based on a physically realistic force field, we show that all the computed models have very flexible terminals of about 200 amino acids each, and a high proportion of helical regions. Properties such as potential energy, radius of gyration, hydrodynamics radius, flexibility coefficient, and solvent-accessible surface are also reported. In a previous conference paper [15] we presented a proof-of-concept investigation into building tertiary structural models of the L protein. In this paper, we present a comprehensive study that includes a detailed physics-based simulation, refinement, and analysis of computed models, as well as how the premise of viral insight connects with computer-based discovery. While this paper focuses on the RVFV L protein, the presented work conveys to a broader community a protocol for the tertiary-structure-modeling discovery of multidomain proteins consisting of thousands of amino acids. In particular, the focus on the RVFV L protein and the ability to obtain structural models for this protein enables our laboratories to better understand viral propagation via further studies of L protein-mediated interactions in viral replication and transcription.

This article is organized as follows. Results are first presented in Section 2, providing a description of the process of selecting the more reliable structure models for the adopted domains and their evaluation. Section 2 also describes the mechanism of assembling the domains together, provides twelve predicted RVFV L protein tertiary structures, and relates to properties showing their structural differences. Section 3 places our findings in context by discussing around several important points. Methodological details are provided in Section 4 by describing the various components of the designed protocol for computing, refining, and evaluating the atomistic structural models of the RVFV L protein. The work is concluded in Section 5. Supplementary Materials provide a zipped file with the atomic coordinates of the twelve RVFV L protein model structures containing all atoms in their relaxed positions and a README file identifying the various models.

## 2. Results

**Figure 1:**
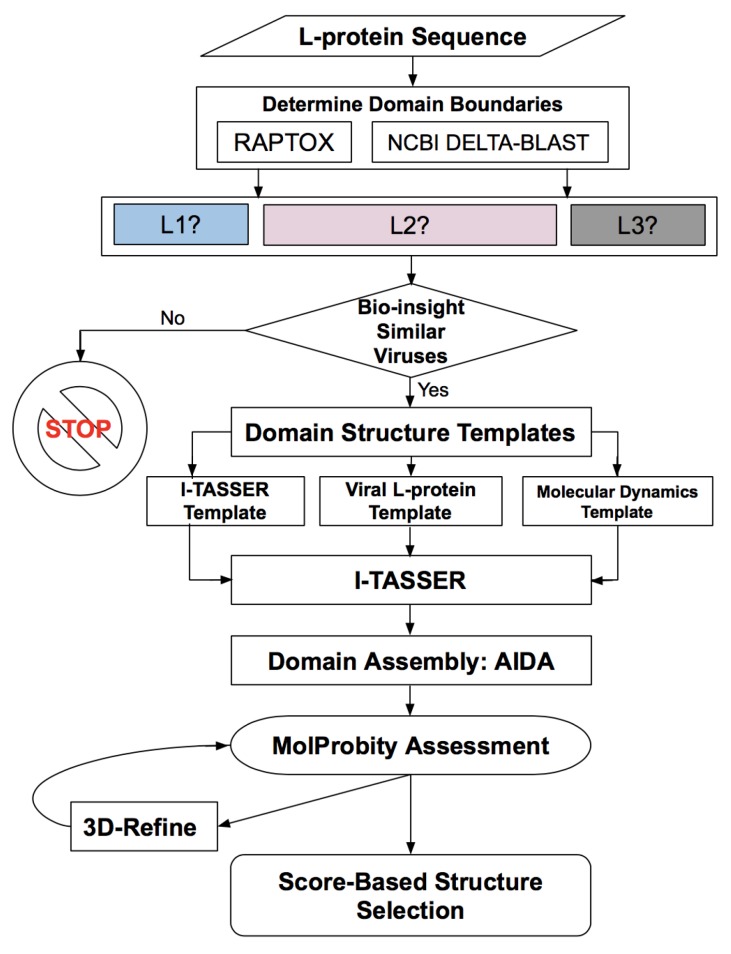
Workflow for determining the protein domains, domain boundaries, domain structure, domain assembly, evaluation and refinement of the Rift Valley fever virus (RVFV) L protein containing 2092 amino acids.

For the RVFV L protein structure determination, we designed a workflow of successive methodologies used for obtaining the protein domains, their boundaries, assembly, refinement, and score-based selection of tertiary structure models, as illustrated in Figure 1. All methods are described in detail in Section 4, while the results obtained from each of the various steps in this workflow are presented below.

### 2.1. Domain Identification

**Figure 2:**
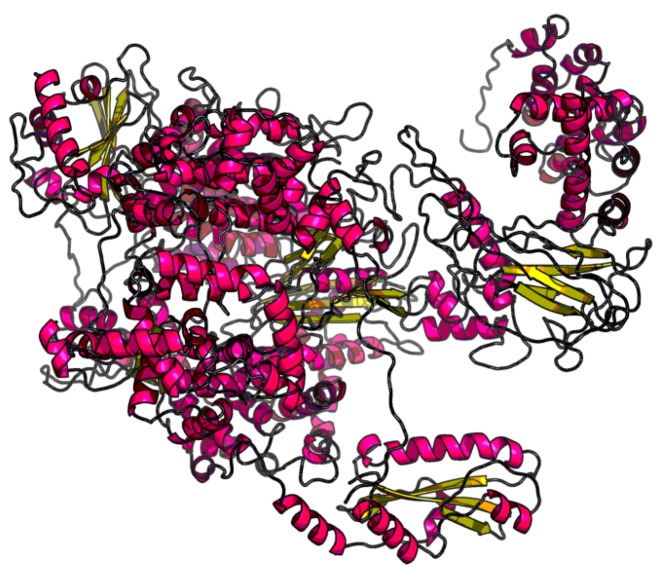
Tertiary structure obtained via RAPTORX for the entire 2092 amino acid sequence of the RVFV L protein. Rendering was performed with Visual Molecular Dynamics (VMD) software [17].

The RAPTORX server predicts a model with three domains, which we refer to as D1, D2, and D3, spanning amino acids (aa) 1–67, 68–1622, and 1623–2092, respectively. The structure predicted for D1 was obtained using the structure under Protein Data Bank (PDB) [16] entry/id 4q84A (sequence identity 9%), while the structure predicted for D2 was from PDB id 5amrA (sequence identity, 12%). The structure predicted for D3 was obtained using two structures, PBD id 4qhpA and 2hpoA (sequence identity, 6%). In addition, RAPTORX predicted that 27 amino acids were disordered; positions 1–3 in the N-terminus (in the D1 domain), and positions 1992–1996 and 2074–2092 in the D3 domain. The full tertiary structure is shown in Figure 2. However, from the analysis described in the next paragraphs, which incorporates information from similar viral L proteins, it is clear that RAPTORX predictions on domain boundaries are unlikely to be correct; hence, no further efforts were invested in this direction.

Following an alternative approach for domain identification, multiple computational possibilities were obtained via NCBI-DELTA BLAST, and several automated servers listed in Section 4.1. For the RVFV L protein, we reconciled the computer-generated possibilities with existing data [18] of the L protein of negative-stranded RNA viruses in the Bunyaviridae family and related Arenaviridae and Orthomyxoviridae families. These viruses indicate the presence of three domains, although information on domain boundaries is only available for the Arenaviridae L proteins [18]. The latter suggests domains for the RVFV L protein that span aa 1–200 in D1, aa 201–1500 in D2, and aa 1051–2092 in D3. We previously investigated this domain identification, and several models were put forward [15]. It was experimentally shown that one stably expressed fragment of the L protein spans aa 1–222 [13]. For this type of experiment, the location of the L-protein truncations were made based on restriction-enzyme locations in the plasmids. Essentially, the truncation points of the L protein were made out of experimental convenience because there were specific DNA sequences at those truncation locations that enabled a restriction enzyme to cut at that location.

**Figure 3:**
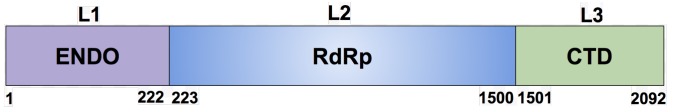
Three domains and boundary determination of RVFV L protein structural models.

Although there is no evidence or established proof for mapping the expressed constructs of L proteins in experiments such as Reference [13] to domains of the protein tertiary structure, in the case of the aa 1–222 fragment, there is a correspondence with a domain boundary based on the mass and charge similarity with domain boundary aa 1–200 of the Arenaviridae L proteins tertiary structures [18]. Therefore, we reconciled all computational models described in what follows with domains and nomenclature: L1 (aa 1–222), L2 (aa 223–1500), L3 (aa 1501–2092), as shown in Figure 3. The L1 domain or ENDO has an endonuclease signature and corresponds to the N-terminus of the protein. The L2 domain, RdRp, is the RNA-dependent RNA polymerase of the protein, and the L3 domain, CTD, corresponds to an extension appended to the C-terminus of the largest subunit of the RNA polymerase that serves as a binding scaffold for numerous nuclear factors. It is worth noting that six fragments of the L protein other than aa 1–222 are stably expressed under the denaturing conditions used in Reference [13]. Those six stably expressed L protein fragments do not correspond in size and boundaries to the tertiary structure domains characterized in this paper as L2 and L3, nor to the domain boundaries of the Arenaviridae L proteins tertiary structures [18].

The full L protein has a mass of 237,955.3 u and a charge of −23 e. The latter is due to its 118 ARG and 134 LYS that have positive charge +1 e plus the 128 ASP and 147 GLU that have a negative charge of −1 e. The three structural domains L1, L2, L3 have masses of 25,303.9, 145,381, and 67,270.4 u, and charges of −16, −8, and +1 e, respectively. Amino acids at the domain boundaries are LEU–ALA for L1–L2 and PRO–ARG for L2–L3.

### 2.2. Modeling the Structure of RVFV L Protein Domains

The next step is determining several plausible structures for each of the three domains, refining, and evaluating them. For that purpose, we identified structural templates for the three domains. Such templates are provided to I-TASSER [19]. This server has the ability to automatically determine a template, an option that we utilize but additionally employ our own templates for the L1 and L2 domains based on known ENDO and RdRp structures of several viral L proteins. For the L1 domain, we also considered the structure of the aa 1–222 fragment extracted from extensive Molecular Dynamics (MD) simulations of the aa 1–250 N-terminus of the RVFV L protein as described in Section 4. For the L3 domain, we used the automatic template from I-TASSER in which the aa 1861–2092 fragment was replaced by the MD optimized structure. We hypothesize that providing biological insight through the templates is crucial for obtaining reasonable structural constructions. Therefore, we prepared domain templates based on known structures of several viral L proteins. Data in Table 1 relate the viral information used in the templates. The Hantaan, La Crosse, and Andes viruses belong to the Bunyaviridae order, same as the RVFV, but are from different families. These viruses are negative-sense single-stranded segmented RNA viruses. The Lassa virus shares these same characteristics, but is in the Arenaviridae family. Meanwhile, the Hepatitis C and Thosea Asigna viruses (both positive-sense) and the Metapneumovirus (negative-sense) single-stranded viruses also do not belong to the Bunyaviridae order. We considered them for the L2 domain because their structures are available and because RdRp is the most conserved L protein domain that shares features of both negative- and positive-sense RNA viruses [20].

Table 1 relates structural evaluation by showing the I-TASSER-provided C-score of the five structural models for the L1 domain, the six models for the L2 domain, and two models for the L3 domain, obtained using I-TASSER with five/six/two different templates. The first row pertains to the I-TASSER automatically selected template (referred to as L1-nt, L2-nt, or L3-nt for the corresponding domains). The next four/five rows for L1/L2 identify templates of structures belonging to several viruses’ L proteins. The notation adopted for these models, L1-(PDB id) and L2-(PDB-id), corresponds to the PDB ID of the virus protein structure used in the template. The L1-MD and L3-nt-MD models contain the structures optimized with MD. Values in bold indicate templates with better C-score. Table 1 lists two additional structural models for domain L3, L3-AIDA and L3-Chimera. These models are created by assembling with AIDA [21] or Chimera [22] the structures of two fragments of L3, the one taken from L3-nt and minimized within AMBER force field (aa 1501–1861) and the other being the MD optimized structure of fragment aa 1862–20192. Properties of these structures, L1 and L3 MD-optimized fragments (aa 1–222 and 1862–2092), and the L3-minimized fragment (aa 1501–1861), are reported in in Table 2.

### 2.3. Assembled L Protein Full-Length Structural Models

In assembling the L1, L2, L3 structural models for building the overall L protein tertiary structure, several domain models are viable based on their C-score. Table 1 lists the C-score for the models considered and highlights in bold the C-score of two models for L1 and three models for L2 that we retained. Additionally, we retained the L1-MD domain model and all four models listed in Table 1 for the L3 domain. This selection reduced the possible L1 + L2 + L3 combinations to 36 assembled structural models. These full-length structural models were evaluated with Molprobity scores and refined with 3DRefine; both techniques are described in Section 4. After refinement, re-evaluation was performed, yielding the 12 tertiary structure models with the lowest MP scores reported in Table 1. Score values are listed as “before min” or “after min” in Table 3, indicating the score obtained before or after further structural relaxation/minimization. This result points out a surprising selection of the L2 structure based on the Thosea Asigna virus, a positive-sense RNA virus from the Permutotetraviridae family and Alphapermutotetravirus genus. Consequently, this virus is not closely related to RVFV. However, what may partially explain our results is the fact that the RdRp domain is the most conserved of the L-protein-possessing features shared by both negative- and positive-sense RNA viruses [20].

**Figure 4:**
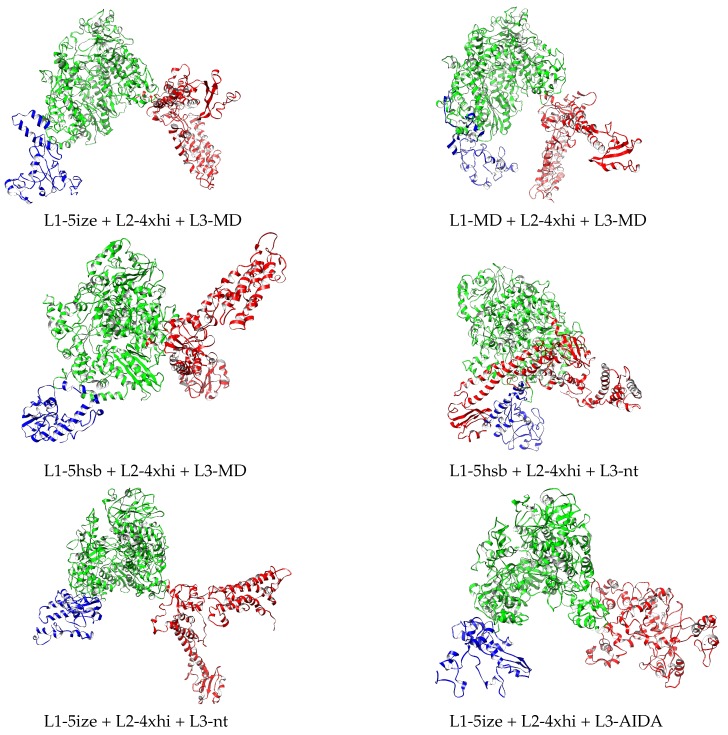
Relaxed structures corresponding to the MP score before and after relaxation in Table 3. L1, L2, and L3 domains depicted in blue, green, and red, respectively. Drawn with Chimera [22].

The best four models according to the MP score are highlighted in bold in Table 3 (before minimization columns) and illustrated in Figure 4. These four models are subjected to new extended refinement, as follows: (i) the structures of the domains in each full-length model are refined with 3DRefine, (ii) the resulting single-domain structures are then assembled with AIDA into a full-length model, and (iii) the AIDA-assembled full-length models are refined again with 3DRefine. Evaluation of the resulting models yields a slight improvement of the MP scores of 3.51 for L1-5ize + L2-4xhi + L3-MD, of the MP scores of 3.49 for L1-MD + L2-4xhi + L3-MD, 3.42 for L1-5hsb + L2-4xhi + L3-MD, and 3.40 for L1-5hsb + L2-4xhi + L3-nt. In order to identify the differences between them, the FATCAT algorithm [23] that optimizes alignment between two structures and minimizes the number of rigid-body movements (twists) around pivot points (hinges) is used. According to this algorithm, these four structures are similar. For example, the L1-5ize + L2-4xhi + L3-MD and L1-MD + L2-4xhi + L3-MD structures are significantly similar, with a p-value of 0.0 (raw score of 5619.35), with identification of 2014/2092 equivalent positions and root-mean-squared deviation (RMSD) of 1.34 Å, with 5 twists.

### 2.4. Energetic Refinement of Full-Length RVFV L-Protein Model Structures and Their Properties

The structural models listed in Table 3 do not contain hydrogen atoms. These are added, and subsequently the 12 structures are relaxed under AMBER force field ff14SB in implicit solvent with pH 7.4 as described in Section 4 for the process of minimization. Supplementary Materials provide the PDB files with the coordinates of all atoms of the twelve relaxed structures. With this energetic refinement, new Molprobity evaluation was performed, with results shown in Table 3, value to the right within each column (after minimization). Improvement was very significant for all twelve structures. More importantly, out of the best four models prior to minimization, only two remained in the group of the best four relaxed structures, L1-MD + L2-4xhi + L3-MD and L1-5hsb + L2-4xhi + L3-MD. The four relaxed structures with lowest MP scores are depicted in Figure 4.

**Figure 5:**
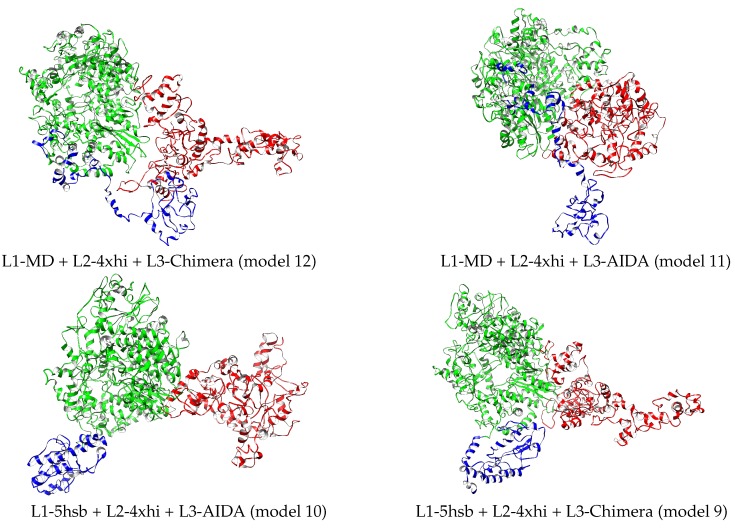
Best four relaxed structures based on L-protein energetics reported in Table 4. L1, L2, and L3 domains depicted in blue, green, and red, respectively. Drawn with Chimera [22].

**Figure 6:**
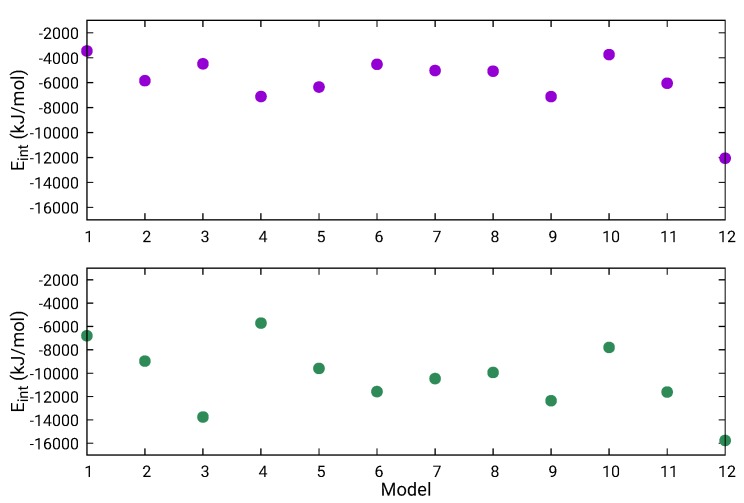
Interaction energy between contiguous domains L1–L2 (magenta) and L2–L3 (green) within the full L protein. Structural models numbered by the order they appear in Table 4.

Considering that the full L protein has 33,287 atoms, an atomistic quantification of the differences between the 12 final models is relevant. Therefore, energetics, size, and shape properties were calculated and are reported in Table 4. The first column gives the potential energy per atom of the L-protein model and indicates that the most stable structure is L1-MD + L2-4xhi + L3-Chimera, followed by L1-MD + L2-4xhi + L3-AIDA, with two other energetically higher structures, L1-5hsb + L2-4xhi + L3-AIDA and L1-5hsb + L2-4xhi + L3-Chimera, separated by ≈0.8/atom kJ/mol. These four models are shown in Figure 5; they contain the MD-optimized fragment of L3 assembled with Chimera or AIDA, but differ in L1. The two models with the lowest energy contain the MD-optimized full L1 domain, while the higher energy ones have this domain based on a fragment from the Andes virus L protein. None of these four models were scored favorably based on the MP score as shown in Table 3. Defining interaction energy between two contiguous domains as $E_{int}=E_{total}-\left(E_{domain}+E_{total-domain}\right)$, the trend of such interaction energy across the twelve structural models is depicted in Figure 6. As illustrated in Figure 6, domains L2–L3 are more strongly bound than domains L1–L2. Although interaction energy has fluctuations across the different models, it is worth noting that the most stable structure, L1-MD + L2-4xhi + L3-Chimera (number 12 in Figure 6) definitely has tertiary domains that are the most strongly bound among the set of predicted models. Additionally, there are interesting trends in the other properties of the twelve structural models related in Table 4.

**Figure 7:**
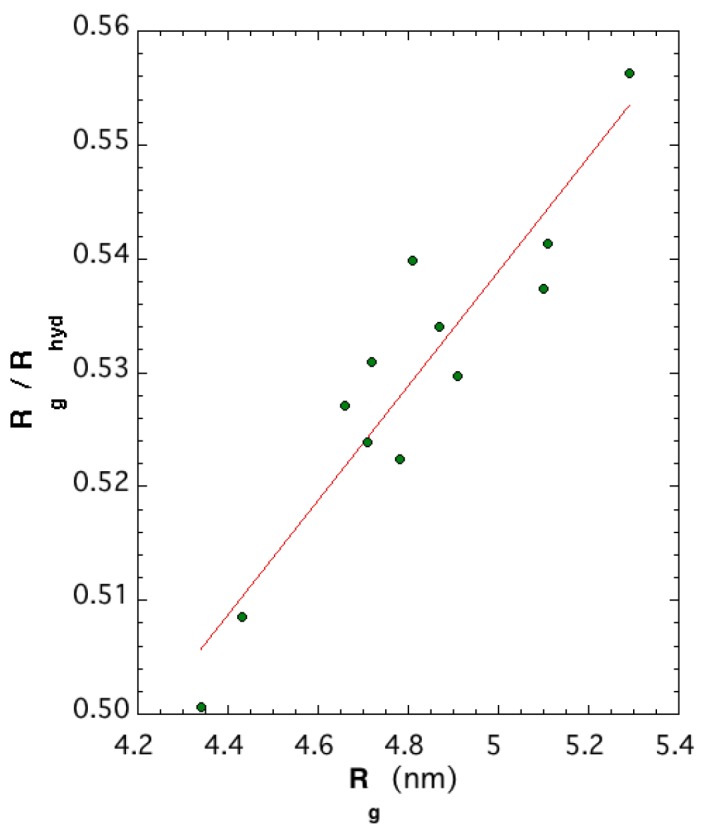
Ratio $R_{g}/R_{hyd}$ versus radius of gyration of the 12 structural models.

A possible measure of protein size is radius of gyration $R_{g}^{2}=\sum_{i=1}^{n}{\left(r_{i}-r_{\mathrm{cm}}\right)}^{2}/n$, where $r_{i}$ are atomic position vectors, $r_{\mathrm{cm}}$ is the center of mass position vector, and n is the number of atoms of the structure. An approximation of the Stokes radius measurable from size-exclusion chromatography is hydrodynamic radius $\frac{1}{R_{hyd}}$=$\frac{1}{n^{2}}$$\sum_{i=1}^{n-1}\sum_{j<i}^{n}\frac{1}{r_{ij}^{2}}$, where $r_{ij}$ are distances between particles *i* and *j*. While $R_{g}$ is slightly more dependent on the structure of the protein of interest than $R_{hyd}$, their ratio $R_{g}/R_{hyd}$ provides information on the molecular shape. The characteristic $R_{g}/R_{hyd}$ value of a globular protein is ≈0.77 or ${\left(3/5\right)}^{1/2}$ [24]. When molecules deviate from globular to nonspherical or elongated structures, then $R_{g}/R_{hyd}$ tends toward values away from 0.77. Figure 7 illustrates that the twelve L protein models are strongly not spherical, with a ratio around 0.53. Correlation between the $R_{hyd}$ of folded or unfolded proteins and the number of residues indicates that models obtained with 2092 residues are consistent with unfolded proteins through empirical equation $R_{hyd}=\left(2.21\pm 1.07\right)\;2092^{0.5\;\pm \;0.02}$ [25]. However, the latter is a formula fitted on smaller-size proteins with at most one-fourth of aa in number. Our result was expected since the three domains retained their own characteristic shape, illustrated in Figure 4 and Figure 5. For these large proteins, there are no established theoretical models for the aggregation of different domain shapes.

Another useful property is flexibility coefficient $C_{n}$, defined in terms of end-to-end distance $R_{e-e}$ and the mean of the *n* backbone bond lengths <l> as $C_{n}={\left(R_{e-e}/<l>\right)}^{2}/n$. For the L protein, <l> = 1.44 ±0.07 Å and n=6275. The values of these properties are reported in Table 4. Because all structural models have the same backbone bonds, $C_{n}$ is driven by values of end-to-end distance; smaller $C_{n}$ values are consistent with more flexible structures since backbones with small bending angles and with flexible bonds would have lower flexibility coefficients [26]. The Chimera-assembled models had the lowest SASA and among the lowest $C_{n}$, indicating that the L1 and L3 domains might have been shielding the L2 domain from the solvent. However, the significantly large flexibility of these two domains is indicative that the 12 structures are snapshots of an overall time-dependent SASA. The last column of Table 4 shows the RMSD between the structure of the model with the lowest PE and the 11 other structures. These RMSDs are substantial, indicating that the models have significant structural differences between them. Models containing the L3 domain assembled with Chimera had the lowest RMSD. This was expected since target structure L1-5hsb + L2-4xhi + L3-Chimera was assembled with Chimera.

## 3. Discussion

This paper shows the complexity involved with obtaining physically realistic structural models of multidomain proteins of thousands of amino acids. As the results demonstrate, while there are several computational approaches for the identification of domains, the building of individual-domain structural models, the assembly of these models into structural models of full-length proteins, and the refinement of such structures must be supplemented with appropriate biological insight for the system at hand. As we convey here for the RVFV L protein, the insight and guiding that known structures of similar viral L-protein domains provide are crucial. This information leverages existing options with regard to domain boundaries and templates for building structural models of various single domains.

An important consideration is the extent to which the built structural models represent the biologically active state of the protein under consideration. In the absence of direct feedback from Cryo TEM, NMR, or previous modeling attempts, this task is nontrivial. Indeed, this is a known problem, referred to as decoy selection in computational structural biology, and research is active but limited to models developed for short single-domain proteins not exceeding 300 amino acids. In ongoing research on the RVFV L protein, we are considering various future avenues for investigating the credibility of the computed models. For example, we are exploring the use of protein-painting for determining the exposed amino acids on the surface of the L protein, which would provide additional biological insight into the tertiary structure of the RVFV L protein. Relating our modeling data with the stable-expression construct of the 1–222 aa segment of Reference [13] ensures that some information on the biological function of the protein under study is embedded in the models besides the insight coming from Arenaviridae L proteins [18]. However, it is worth noting that SDS–PAGE experiments do not give any structural protein information. In our preceding conference article [15] we put forward two tertiary structure models, L1-5ize + L2-4xhi + L3-nt and L1-5hsb + L2-4xhi + L3-nt, with the same viral input of Models 2 and 3 in Table 4 but with their L1 and L2 domains spanning aa 1–200 and aa 201–1500, respectively. The RMSD between the previous models and the current ones was 0.75 and 0.55 nm for the L1 and L2 domains, respectively. Considering that the L1 domain is composed of 3514 atoms and the L2 domain has 20,332 atoms, the RMSD between the structure of the two types of L1 was very small and had a negligible effect on the L2 domain structure. This comparison indicates that the structure of the L1 and L2 domains of Models 2, 3, 4, 6, 7, 8, 9, 10 (Table 4) would have been very similar to models built with L1 domains spanning aa 1–200, and L2 domains spanning aa 201–1500.

Moreover, we did not exclude that more than three domains may exist in the RVFV L-protein tertiary structure. In that respect, the computed structural models would not fully capture the biologically active state of the RVFV L protein. However, models put forward in our work provide useful snapshots of the possible equilibrium flexibility of the L protein, particularly as it relates to protein termini. The results obtained via RAPTORX point to disorder predicted for amino acids in the N-terminus (L1 domain) and C-terminus (L3 domain). More importantly, these findings were further corroborated by our MD simulations, which indicated that the two end terminals of the RVFV L protein, at the studied thermodynamic conditions, are quite flexible and may adapt their structure upon the environment in which they are immersed under physiological conditions.

Given the sparsity of information on the RVFV L protein and the lack of a crystal structure, the findings presented here on the structural models of the RVFV L protein tertiary structure facilitate protein–protein interaction studies and drug-discovery efforts. In fact, viral polymerases make one of the most heavily targeted viral proteins for the development of antivirals [14]. Some well-known examples include Acyclovir, which is a herpesvirus DNA polymerase inhibitor, and AZT that inhibits HIV reverse transcriptase. For RVFV, there has been some success with using nucleoside inhibitors, such as favipiravir, to prevent pathogenesis in small-animal models of disease [27]. Thus, continued research on the L-protein tertiary structure is critical to enable the development of more selective and efficacious drugs for RVFV treatment.

Furthermore, given the high-sequence identification of the RVFV L protein and similar RdRp of related viruses, the L-protein structural models may also serve as valuable templates for improving the structural characterization of other polymerases. In this respect, the organization of eventual aa groups [28] within the well-identified sequence of 2092 aa in the RVFV L protein may spearhead where mutations might affect function, and may unravel characteristic patterns to the L proteins of specific viruses. It is worth mentioning that while this work focuses on the RVFV L protein, the presented findings provide a methodological roadmap for the tertiary-structure modeling of large multidomain proteins for the broader community.

## 4. Materials and Methods

Homology or comparative modeling is the most reliable way to predict the tertiary structure of a given amino acid sequence. Protein structures are more conserved than protein sequences among homologs, but sequences falling below a 20% sequence identity can have very different structures [29]. Therefore, a first step is searching for homologous proteins to the full 2092 aa long sequence of the RVFV L protein with at least 20% sequence identity in the PDB. Using NCBI BLASTP [30] revealed that there were no sequences homologous with the entire RVFV L-protein sequence in the PDB. However, searching with NCBI SMART BLAST [31] for homologous sequences in the landmark database (contains only sequences) reveals that the RVFV L protein sequence is homologous with three viral polymerases from the same viral family, the Ambe, Joa, and Salobo viruses (sequence identities range from 60% to 61%). Unfortunately, none of these polymerases have known tertiary structures.

In the absence of a homologous sequence with a known structure, the goal then becomes to identify possibly conserved segments of the entire sequence with known structures, and then assembling together structures of such segments to obtain a model for the entire sequence. Two directions can be pursued: using fully automated servers, or controlling the definition of domains and the identification of template structures for the domains in a way that incorporates additional biological insight about the system at hand.

Servers such as RAPTORX [32] support remote homology recognition and protein threading, and implement a fully automated process of structure prediction for long protein sequences. Specifically, RAPTORX explores whether the target sequence consists of multiple domains or is a single folding unit. In the former case, the sequence is partitioned into domains, and structures in the PDB that are remotely homologous to the domains are identified and used as templates. We note that RAPTORX distinguishes itself from other structure-modeling servers by the quality of the alignment between the target sequence and one or more distantly related templates (especially those with sparse sequence profiles), and by a novel nonlinear scoring function and a probabilistic-consistency algorithm. RAPTORX has been reported to deliver high-quality structural models for many targets with only remote homology templates [33].

### 4.1. Domain Identification

Domains may be separately identified from the structure-prediction protocol by incorporating biological insight. There are multiple options available to researchers for domain identification. For example, one can identify conserved regions via NCBI DELTA-BLAST [34] in knowledge-based databases, such as the pfam database [35]. Alternatively, one can make use of domain-identification servers such as ThreaDomEX [36], Dobo [37], DomCut [38], and DomPred [39]. Results from these two different approaches and different automated servers may be quite different, both in the number of predicted domains and in their boundaries. Biological insight can be used to finalize the domains. For example, information may be available from the biological studies of related proteins. Specifically for the RVFV L-protein, information is available on L proteins from other related viruses, such as the arenavirus and other arenaviridae, which suggests the presence of three domains. This biological insight can be used to reconcile findings of many servers regarding the number of domains and domain boundaries, as we do here (and show in Section 2 for the RVFV L protein).

### 4.2. Domain-Structure Modeling

For each set of the identified domains, a tertiary structure can be modeled. While, in principle, template-free methods, such as Rosetta and Quark can be used, when the domains exceed 200 amino acids in length, a template-free strategy is not viable. Instead, threading-based methods, such as I-TASSER [19] can be used. Such methods automatically identify structural templates based on sequence-environment alignments, and this default setting can be used to obtain structural models for the identified domains. While such models are ranked based on a statistical-based structural *goodness* score, one cannot only ely on such a score to determine which structure is the correct one for the domain under investigation. Instead, more options need to be considered. Biological insight can be leveraged to identify structural templates that are not obvious to the automated setting in I-TASSER. Specifically designed templates enhance the probability of finding relevant structural models for a domain. In addition, a molecular-dynamics-based setting can be considered, where simulations of long trajectories can be leveraged to obtain low-energy structural models for a domain. Moreover, each of the structural templates obtained via these different approaches can further be energetically refined/minimized.

### 4.3. All-Atom Molecular-Dynamics Investigation, Structural Relaxation, and Energetics Evaluation

We conducted all-atom MD simulations in implicit solvent at a constant pH = 7.4 [40] using the AMBER software package [41,42,43]. The interactions were modeled with the all-atom ff14SB force field [44] and the generalized Born model for the implicit solvent [45]. The selected pH matched the wet-lab buffer solution of the L protein. Two terminal portions of the L protein, peptides 1–250 aa and 1858–2092 aa, were simulated at a constant temperature of 310 K via the Langevin thermostat with a collision frequency of 5 ps${}^{-1}$ and a time step of 1 fs. The equilibration stage implemented a round-robin [46] approach of segmenting each system in smaller portions, equilibrating each of them, and then rebuilding the system for the complete domain for 10 ns at a time. This acceleration strategy was very effective, allowing to faster reach the production stage. The production stage was composed of a collection of 20 trajectories of 5 ns for a total run time of 100 ns for each peptide. During the MD production stage, the four end aa of each peptide connecting to the bulk of the L protein (aa 247–250 and 1858–1861) were restrained and kept fixed in space during all runs. This strategy was adopted for emulating the fact that the two end peptides are joint to a large mass and not free of rigidly translating or rotating. Each peptide 5 ns trajectory was started with random velocities and the last configuration of the previous run except for the last 20 ns. For the latter, runs were initiated from the minimized final configuration of the previous run and assigned random velocities. The MD-optimized peptides, provided as templates for I-TASSER or to build the final structural models, were selected from a configuration with low energy of the last 5 ns run of each terminus peptide. We note that the structure for the L1 domain, aa 1–222, is a portion of the MD-optimized larger peptide aa 1–250.

The AMBER package allows for the relaxation of a structure via minimization of the molecular potential energy. This approach was used for obtaining the properties of relaxed full L-protein structures presented in Table 4 and their Molprobity scoring reported in Table 3. Specifically, potential energy PE under the AMBER ff14SB force field in an implicit solvent at the constant pH of Table 4 is considered as an evaluation of the different relaxed structural models. Each of these models has 2092 aa and the same sequence. Models with the lowest potential energy are the most stable and, therefore, have a higher probability of being realized. The relaxation approach was also used for minimizing the L3 portion spanning aa 1501–1861 in several templates fed to I-TASSER. Properties of such a portion of L3 are reported in Table 2. The steepest descent method was employed for the minimizations with a convergence criterion for gradients of 0.0001 kcal/mol-Å.

### 4.4. Assembling Structural Models of Single Domains into a Full-Length Tertiary Structure

We considered AIDA [21] and Chimera [22] as methods for assembling together the various models obtained for each domain. AIDA computes the best spatial arrangement of (provided structural models of) domains under an optimization process that seeks to lower the potential energy of knowledge-based ab initio terms [21]. In Chimera, single-domain models can be connected via the *join* function. Default values are used to form a C–N peptide bond (to assemble structural models) (C–N length = 1.33Å,C${}_{\alpha }$-C–N–C${}_{\alpha }$ dihedral angle ω = $180.0^{{}^{\circ}}$,C–N–C${}_{\alpha }$-C dihedral angle ϕ = $-120.0^{{}^{\circ}}$).

### 4.5. Tertiary-Structure Refinement

Refinement methods span a variety of methodologies, such as 3DRefine [47], Galaxy [48], ReFold [49], PREFMD [50], and KoBaMIN [51]. However, Galaxy is also limited to sequences less than 1000 amino acids in length, and the KoBaMin web server (source code not available to researchers) is currently not responsive. As a result, all nonminimization refinements related in Section 2 were conducted via 3DRefine.

### 4.6. Tertiary-Structure Evaluation

The resulting full-length models could be ranked via various metrics to reveal a few top structural models that may comprise the native state of the protein target of interest. Evaluation can consider several metrics. Structural models of the entire multidomain protein (obtained by assembling structural models of the constitutive domains) can be evaluated via evaluation metrics utilized in CASP to determine the quality of computed structural models. Specifically, we considered Molprobity scores, such as Clash score, Rot-out, Ram-out, Ram-fv, and MP score [52]. Clash score is the number of severe atomic clashes (with an overlap of <0.4 Å) per 1000 atoms. Rot-out is the rotamer outlier score that measures the percentage of side-chain configurations classified as rotamer outliers. Ram-fv is the Ramachandran-favored score measuring the percentage of backbone Ramachandran configurations classified as outliers. The MP score combines these scores into a mathematical expression as:

$$\begin{array}{c} \text{MP-score}=0.426\ln(1+\text{Clash-score})+0.33\ln(1+\max(0,\text{Rot-out}-1))\\ +0.25\ln(1+\max(0,(100-\text{Ram-fv}))-2))+0.5. \end{array}$$

## 5. Conclusions

Leveraging a battery of methodologies from computational molecular biology and bioinformatics, this work predicts twelve plausible tertiary-structure models of the 2092 aa long RVFV L protein. Although some used approaches have become routine methods for finding structural models in the life sciences, the ability to insert known structural information from similar viruses and details of realistic atomic interactions in appropriate thermodynamics conditions leads to a group of tertiary-structure models of structurally distinct functional states. Inclusion of the dynamics information through MD for the L-protein termini predicts that this L protein is not a static macromolecule and that structural flexibility must be taken into account. So far, exact correspondence between our experimentally detected expressed fragments and the three computationally predicted domains is not perfect. However, it is unknown if the fragmentation evidenced through expression constructs can be mapped into the domains of a multiple-domain protein. This is an open field of investigation.

The twelve models of the RVFV L-protein tertiary structure have sizes with radii of gyration between 4.34 and 5.29 nm, and SASAs between 748 and 845 nm${}^{2}$. The tertiary structures are far from being globular as evidenced by ratio $R_{g}/R_{hyd}\approx$ 0.53; they resemble a dense clusterlike region (L2) with two elongated leglike regions (L1, L3). Based on energetics, the more stable structures had the two leglike domains structures optimized by extensive MD simulations under the AMBER ff14SB force field in implicit solvent and a pH of 7.4. The most stable structure possessed the most strongly bound domains, with the interaction energy at the L1–L2 domain boundary being weaker than the interaction energy at the L2–L3 boundary. The L2 domain originated from a template based on the L protein of the Thosea Asigna virus that was selected as the best for all twelve tertiary-structure models. This is a surprising result because this virus belongs to a different order than the RVFV and is positive-sense-stranded. In future investigation, we plan to extend MD modeling and simulation to portions of the L2 domain or the full domain. The ensemble of twelve tertiary-structure models put forward in this work was a challenge. The ideas described can be easily applied to the prediction schema of other viral L proteins, and it might well be expected that, by adopting the workflow utilized here, other tertiary-structure prediction approaches would show measurable improvements. Our expectation is that the set of structural models of the tertiary structure put forward here would encourage relevant experimentation tending to elucidate their veracity. 

## Figures and Tables

**Table 1:** Structural models considered for the L1, L2, and L3 templates provided to I-TASSER [19]. C-score values in bold are the best for domains L1 and L2.

PDB id	Organism	C-Score	PDB id	Organism	C-Score	Model	Description	C-Score
L1-nt	-	−4.32	L2-nt	-	−0.09	L3-nt	I-TASSER model	−1.68
L1-4miw	Lassa virus	−1.55	L2-5amq	La Crosse	**0.07**	L3-nt-MD	L3-nt with MD _aa 1861–2092_	−1.95
L1-5ize	Hantaan virus	**−0.97**	L2-5amr	La Crosse	−0.09	L3-AIDA	L3-nt-MD, AIDA	-
L1-5hsb	Andes virus	**−0.74**	L2-1yuy	Hepatitis C	−0.05	L3-Chimera	L3-nt-MD, Chimera	-
L1-5j1n	Lassa virus	−1.45	L2-4xhi	Thosea Asigna	**0.04**			
L1-MD	-	-	L2-4ucy	Metapneu- movirus	**0.17**			

**Table 2:** Properties of the molecular-dynamics (MD)-optimized structures of domains L1 and L3: potential energy per atom PE, radius of gyration $R_{g}$, end-to-end distance $R_{e-e}$, and maximum radius from center of mass $R_{max}$.

Domain Segment	PE (kJ/mol)	$R_{e-e}$ (nm)	$R_{g}$ (nm)	$R_{\max}$ (nm)
L1-MD${}_{1–222}$	−8.62	6.66	3.05	6.03
L3${}_{1501–1861}$	−7.57	7.23	2.45	5.47
L3-MD${}_{1862–2092}$	−8.70	3.89	2.97	5.11

**Table 3:** Molprobity evaluation of full-length L protein structures refined with 3DRefine, before and after structural relaxation. The best MP-scores are given in bold.

	MP-Score		Clash-Score		Rot-Out		Ram-Out		Ram-fv	
**Model**	Before min	After min	Before min	After min	Before min	After min	Before min	After min	Before min	After min
L1-5ize + L2-4xhi + L3-nt	3.75	**2.48**	74.1	1.89	7.91	8.52	8.42	7.22	80.53	72.16
L1-5ize + L2-4xhi + L3-MD	**3.70**	2.52	73.34	1.95	7.53	9.68	7.85	6.96	82.54	78.54
L1-5ize + L2-4xhi + L3-AIDA	3.79	**2.49**	73.36	1.71	8.82	9.18	9.76	8.40	80.05	71.19
L1-5ize + L2-4xhi + L3-Chimera	3.91	2.60	88.81	2.04	9.84	10.95	10.07	7.47	79.58	70.82
L1-5hsb + L2-4xhi + L3-nt	3.76	2.64	77.25	2.61	8.02	10.12	8.18	7.27	81.15	72.94
L1-5hsb + L2-4xhi + L3-MD	**3.73**	**2.50**	74.62	1.98	8.23	9.07	7.70	7.22	83.16	73.20
L1-5hsb + L2-4xhi + L3-AIDA	3.78	2.56	77.23	2.16	8.23	9.40	9.33	6.96	80.19	71.39
L1-5hsb + L2-4xhi + L3-Chimera	3.90	2.56	89.62	2.13	9.36	9.13	9.73	7.89	79.29	69.43
L1-MD + L2-4xhi + L3-nt	**3.73**	2.60	72.28	2.16	8.02	10.90	7.94	6.24	81.53	72.37
L1-MD + L2-4xhi + L3-MD	**3.70**	**2.49**	76.02	1.86	7.42	9.18	7.42	6.86	83.43	72.89
L1-MD + L2-4xhi + L3-AIDA	3.77	2.53	72.81	1.89	8.66	9.79	9.04	7.68	80.57	71.75
L1-MD + L2-4xhi + L3-Chimera	3.90	2.57	90.22	2.28	9.63	9.07	10.16	8.45	80.30	70.36

**Table 4:** Potential energy per atom PE, radius of gyration $R_{g}$, hydrodynamic radius $R_{hyd}$, flexibility coefficient $C_{n}$, end-to-end distance $R_{e-e}$, and solvent-accessible surface area SASA of RVFV L protein structural models. Values correspond to relaxed structures after minimization. Models provided in decreasing PE order. Last column lists the root-mean-squared deviation (RMSD) of each model with respect to the most energetically stable model, L1-MD + L2-4xhi + L3-Chimera. Bold values indicate the structures of lowest PE.

Model		PE (kJ/mol)	$R_{g}$ (nm)	$R_{\mathrm{hyd}}$ (nm)	$C_{n}$	$R_{e-e}$ (nm)	SASA (nm${}^{2}$)	RMSD (nm)
1	L1-MD + L2-4xhi + L3-nt	−6.664	5.10	9.49	0.05 ± 0.01	2.58	845.0	3.15
2	L1-5ize + L2-4xhi + L3-nt	−6.710	5.29	9.51	2.05 ± 0.23	16.27	832.4	3.55
3	L1-5hsb + L2-4xhi + L3-nt	−6.751	4.34	8.67	0.25 ± 0.03	5.64	816.0	3.37
4	L1-5ize + L2-4xhi + L3-AIDA	−6.762	4.91	9.27	1.61 ± 0.18	14.33	802.8	3.74
5	L1-MD + L2-4xhi + L3-MD	−6.781	4.78	9.15	0.17 ± 0.02	4.71	826.7	3.14
6	L1-5ize + L2-4xhi + L3-MD	−6.791	5.11	9.44	0.48 ± 0.05	7.88	840.0	3.49
7	L1-5hsb + L2-4xhi + L3-MD	−6.792	4.87	9.12	1.78 ± 0.19	15.04	801.5	3.55
8	L1-5ize + L2-4xhi + L3-Chimera	−6.799	4.66	8.84	0.24 ± 0.03	5.60	759.8	1.85
9	L1-5hsb + L2-4xhi + L3-Chimera	**−6.820**	4.71	8.99	0.54 ± 0.06	8.41	742.8	1.50
10	L1-5hsb + L2-4xhi + L3-AIDA	**−6.833**	4.72	8.89	1.72 ± 0.18	14.80	771.0	3.31
11	L1-MD + L2-4xhi + L3-AIDA	**−6.901**	4.43	8.71	0.66 ± 0.08	9.26	787.3	2.54
12	L1-MD + L2-4xhi + L3-Chimera	**−6.919**	4.81	8.91	0.22 ± 0.02	5.28	747.6	0.00

## Supplementary Materials

Available online is a zipped directory containing a README file identifying the structural-model PDB files containing the coordinates of all atoms of the relaxed structures.

## Abbreviations

The following abbreviations are used in this manuscript:

aa	amino acid
AZT	Azidothymidine
CASP	Critical Assessment of Protein Structure Prediction
CDC	Centers for Disease Control and Prevention
Cryo TEM	Transmission electron cryomicroscopy
FDA	U.S. Food and Drug Administration
HIV	Human immunodeficiency virus
lRMSD	least root-mean-squared-deviation
MD	Molecular Dynamics
NMR	Nuclear magnetic resonance
NP	Nucleoprotein
PAGE	Polyacrylamide gel electrophoresis
PDB	Protein Data Bank
PE	Potential energy
RdRp	RNA dependent RNA polymerase
RMSD	Root-mean-square deviation
RVFV	Rift Valley Fever Virus
SASA	Solvent Accesible Surface Area
SDS	Sodium dodecyl sulfate
USDA	U.S. Department of Agriculture
