# Supplementary material for: Modeling the Tertiary Structure of the Rift Valley Fever Virus L Protein
Source: Molecules. 2019 May 7;24(9):1768. doi: 10.3390/molecules24091768 (PMC6539450; doi:10.3390/molecules24091768)
Supplement: Supplementary file 1 [file molecules-24-01768-s001.zip › README_Supplem_Mat_Gogovi_etal.pdf]

Supplementary Material

Modeling the Tertiary Structure of the Rift Valley Fever Virus L protein

Gideon K. Gogovi, Fahad Almsned, Nicole Bracci, Kylene Kehn-Hall, Amarda Shehu, Estela Blaisten-Barojas

George Mason University, Fairfax, VA 22030, USA

1 Coordinates of Twelve Rift Valley Fever Virus L protein Structural Models

The table below shows the structural models of the RVFV L protein that correspond to each PDB file provided in the zipped directory.

PDB	STRUCTURAL MODEL NAME
protein_min_1.pdb	L1-5ize+L2-4xhi+L3-nt
protein_min_2.pdb	L1-5ize+L2-4xhi+L3-MD
protein_min_3.pdb	L1-5ize+L2-4xhi+L3-AIDA
protein_min_4.pdb	L1-5ize+L2-4xhi+L3-Chimera
protein_min_5.pdb	L1-5hsb+L2-4xhi+L3-nt
protein_min_6.pdb	L1-5hsb+L2-4xhi+L3-MD
protein_min_7.pdb	L1-5hsb+L2-4xhi+L3-AIDA
protein_min_8.pdb	L1-5hsb+L2-4xhi+L3-Chimera
protein_min_9.pdb	L1-MD+L2-4xhi+L3-nt
protein_min_10.pdb	L1-MD+L2-4xhi+L3-MD
protein_min_11.pdb	L1-MD+L2-4xhi+L3-AIDA
protein_min_12.pdb	L1-MD+L2-4xhi+L3-Chimera

2 Rift Valley Fever Virus L Protein Expression Transfections and Western Blot

Previous wet-laboratory investigation has indicated that various fragments of L protein are stably expressed in cell culture [1]. In the experiment described here the L protein expression constructs were a kind gift from Dr. Shinji Makino and have been previously described [1]. To reconfirm these results, we transfected BSR-T7 cells with protein expression constructs of L polymerase and assayed for protein expression via western blot. All of the L protein segments were readily detected with GFP-L CS (aa 1219-2091) showing the highest expression levels as depicted in Fig. 1. The full length L protein, included as a control, was also observed to be stably expressed, but at lower levels as compared to the protein fragments (compare lane 1 to lanes 2-8). Overall, results indicate that previously published fragmentation pattern of L protein is reproducible since it can be expressed independently without compromising protein stability. In this wet-lab experiment, the expression plasmids contain GFP to enable L protein detection and are driven by T7-polymerase. BSR-T7 cells are stably

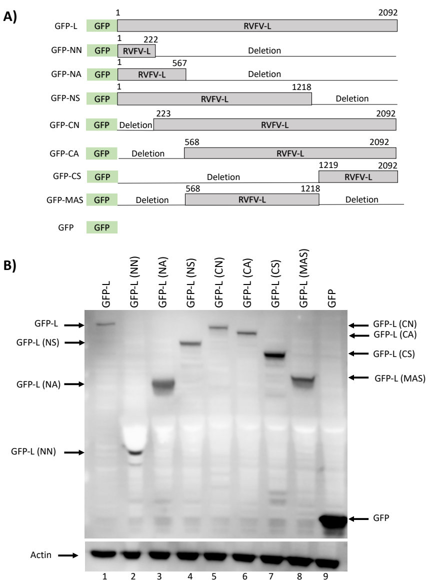

Figure 1: A) Diagram of the expression constructs used. All constructs contain GFP to enable detection. B) BSR-T7 cells were transfected with pT7-IRES-GFP-L, pT7-IRES-GFP-L (NN), pT7-IRES-GFP-L (NA), pT7-IRES-GFP-L (NS), pT7-IRES-GFP-L (CN), pT7-IRES-GFP-L (CA), pT7-IRES-GFP-L (CS), pT7-IRES-GFP-L MAS or pT7-IRES-GFP. Twenty-four hours post-transfection protein lysates were collected and western blot analysis performed using anti-GFP and anti-actin antibodies.

.

transfected with T7-polymerase and were maintained as described [2]. BSR-T7 cells ($3.5x10^5$ cells/well in a 6-well plate) were transfected with 15 μl TransLT1 Reagent (Mirus, Cat # MIR 2304) and 5 μg of pT7-IRES-GFP-L, pT7-IRES-GFP-L (NN), pT7-IRES-GFP-L (NA), pT7-IRES-GFP-L (NS), pT7-IRES-GFP-L (CN), pT7-IRES-GFP-L (CA), pT7-IRES-GFP-L (CS), pT7-IRES-GFP-L MAS or pT7-IRES-GFP according to the manufacturer instructions. Protein lysates were collected twenty-four hours post-transfection with Blue Lysis Buffer [Mixture of 20 ml T-PER reagent (Thermo Scientific Pierce), 30 ml 2 $\times$ Tris-glycine SDS sample buffer (Invitrogen), 1.3 ml 1M DTT, 200 μl 0.5M EDTA pH 8.0, 80 μl 0.1M Na3VO3, 400 μl 0.1M NaF and 1 complete protease inhibitor cocktail (1 $\times$ Halt cocktail, Pierce] and boiled for 10 min. Protein lysates (30 μg) were separated by SDS-PAGE and western blot performed as described [2] using anti-GFP (Abcam, Cat # ab290) and anti-actin antibodies.

3 References

1 Zamoto-Niikura, A.; Terasaki, K.; Ikegami, T.; Peters, C.J.; Makino, S. Rift Valley Fever Virus L Protein Forms a Biologically Active Oligomer. *J. Virol.* 2009, 83, 12779-12789.

2 Benedict, A.; Bansal, N.; Senina, S.; Hooper, I.; Lundberg, L.; de la Fuente, C.; Narayanan, A.; Gutting, B.; Kehn-Hall, K. Repurposing FDA-approved drugs as therapeutics to treat Rift Valley fever virus infection. *Front. Microbiol.* 2015, 6, 676.
